# Supplementary material for: Combining Linkage and Association Mapping Approaches to Study the Genetic Architecture of Verticillium Wilt Resistance in Sunflower
Source: Plants (Basel). 2025 Apr 11;14(8):1187. doi: 10.3390/plants14081187 (PMC12030505; doi:10.3390/plants14081187)
Supplement: Supplementary file 1 [file plants-14-01187-s001.zip › Figure S2.pdf]

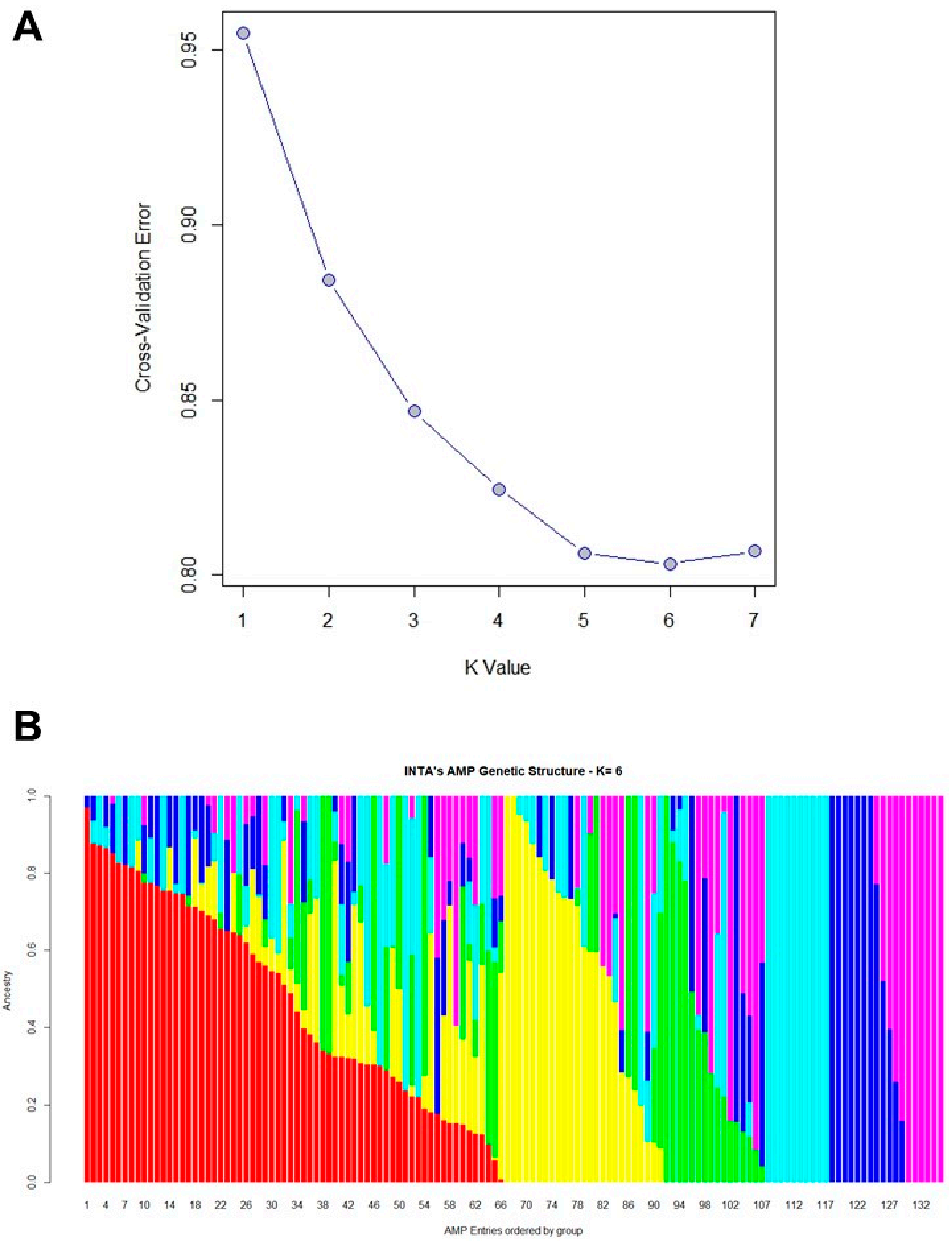

Figure S2. A: Optimal number of genetic clusters using the cross-validation procedure implemented by the ADMIXTURE program. **B:** Population structure inference using the ADMIXTURE program. Each column represents a genotype and the stacked colored bars the proportion of ancestry lineages that compose them.
