# Supplementary material for: Combining Linkage and Association Mapping Approaches to Study the Genetic Architecture of Verticillium Wilt Resistance in Sunflower
Source: Plants (Basel). 2025 Apr 11;14(8):1187. doi: 10.3390/plants14081187 (PMC12030505; doi:10.3390/plants14081187)
Supplement: Supplementary file 1 [file plants-14-01187-s001.zip › Figure S3.pdf]

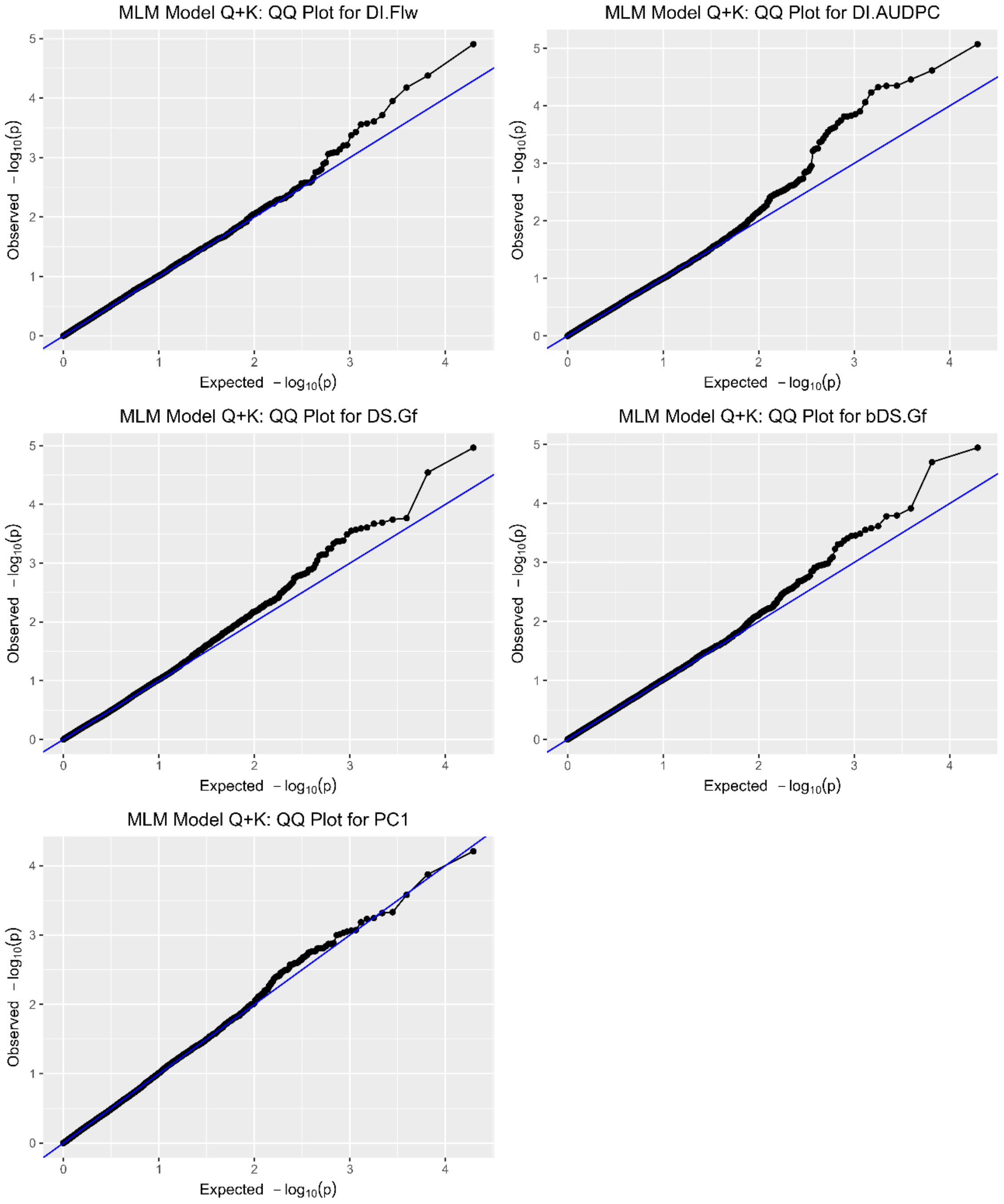

Figure S3. QQ plots of the Mixed Linear Models implemented for each disease descriptor (DI.Flw, DI.AUDPC, DS.Gf, bDS.Gf and PC1).
