# Supplementary material for: Combining Linkage and Association Mapping Approaches to Study the Genetic Architecture of Verticillium Wilt Resistance in Sunflower
Source: Plants (Basel). 2025 Apr 11;14(8):1187. doi: 10.3390/plants14081187 (PMC12030505; doi:10.3390/plants14081187)
Supplement: Supplementary file 1 [file plants-14-01187-s001.zip › Figure S7.pdf]

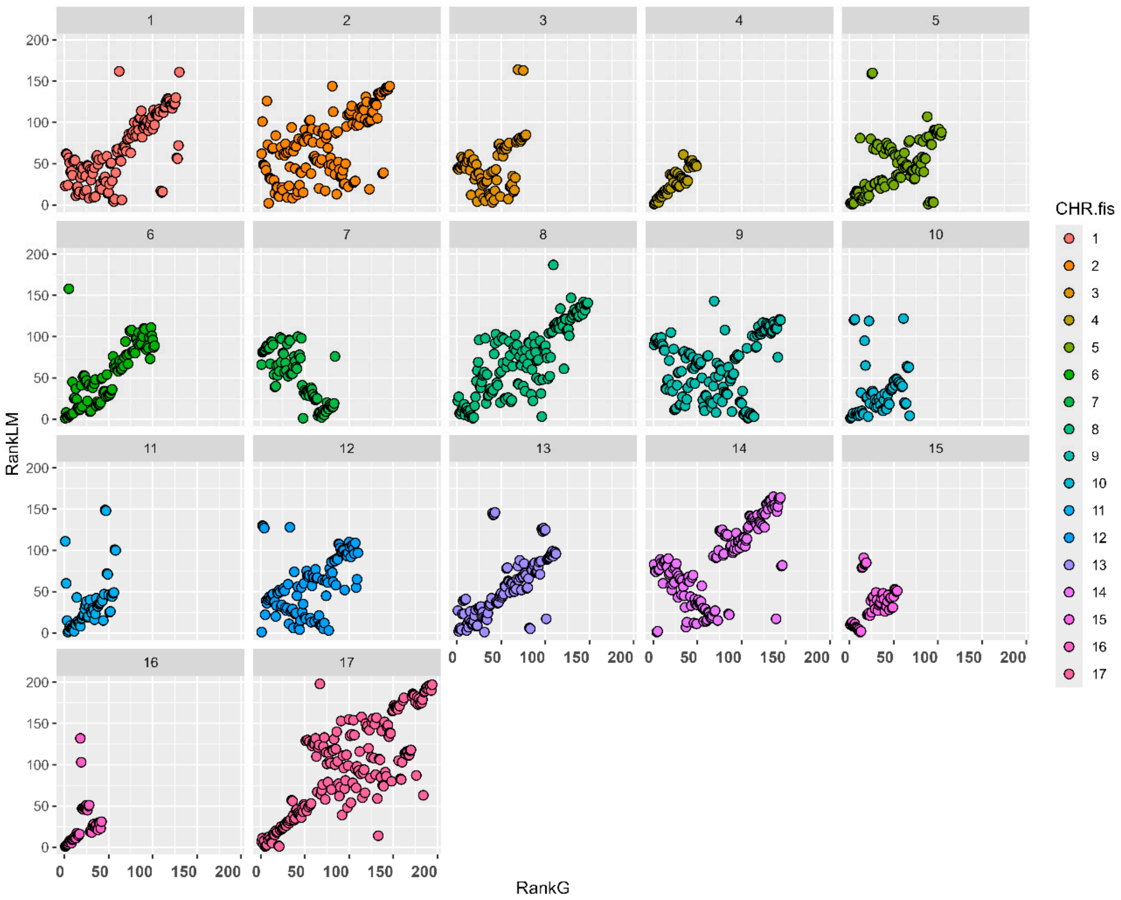

Figure S7. Collinearity between the SNPs distribution of the linkage map obtained from the BMP population and the reference genome (HanXRQ.v1)
