# Supplementary figures and images for: Combining Linkage and Association Mapping Approaches to Study the Genetic Architecture of Verticillium Wilt Resistance in Sunflower
Source: Plants (Basel). 2025 Apr 11;14(8):1187. doi: 10.3390/plants14081187 (PMC12030505; doi:10.3390/plants14081187)

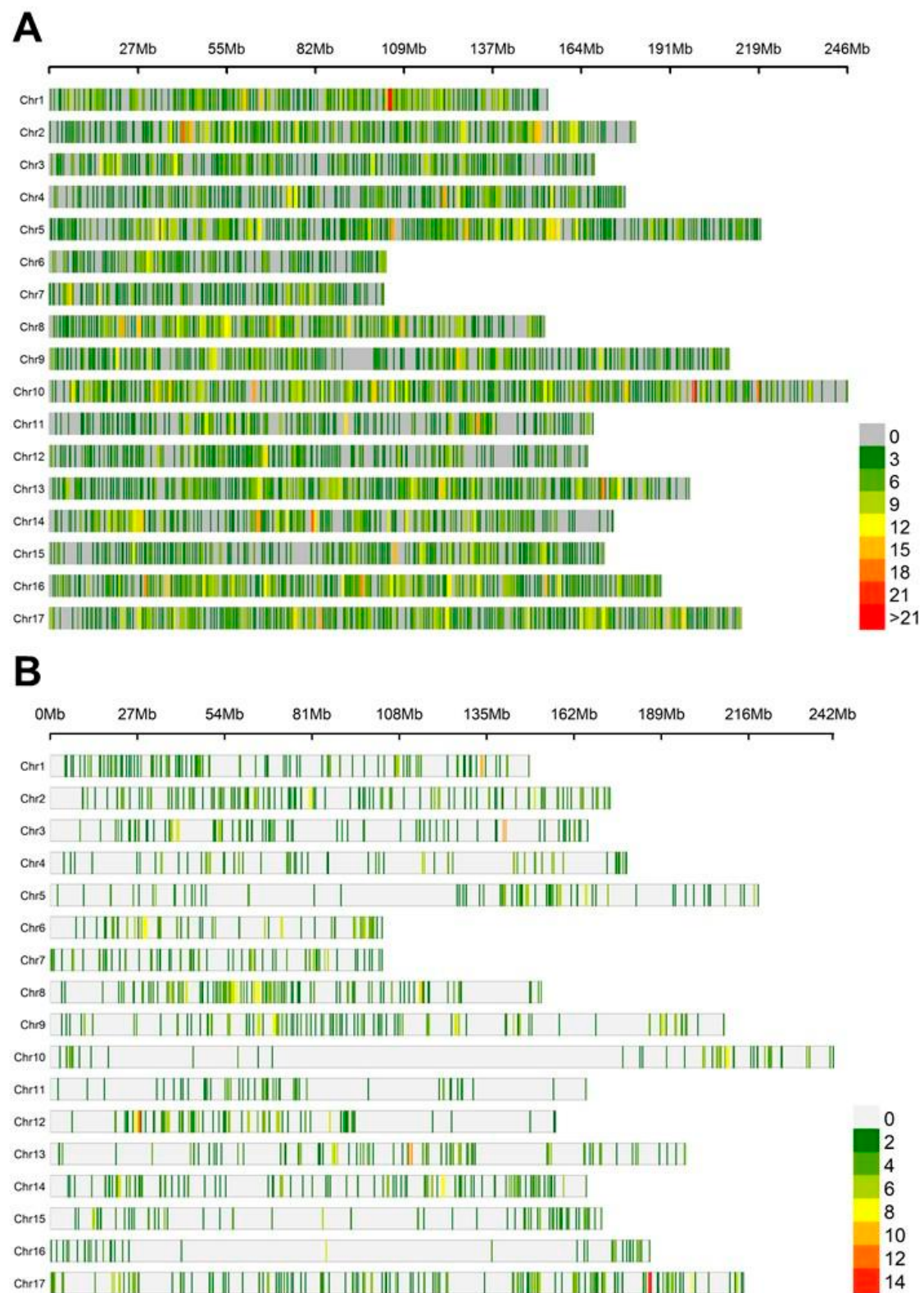

**Figure S5.** SNPs density within 1 Mb window size for both mapping populations. A: AMP. B: BMP.

Supplement: Supplementary file 1 [file plants-14-01187-s001.zip › Figure S5.pdf]
